# Variations in Physiology and Multiple Bioactive Constituents under Salt Stress Provide Insight into the Quality Evaluation of Apocyni Veneti Folium

**DOI:** 10.3390/ijms19103042

**Published:** 2018-10-05

**Authors:** Cuihua Chen, Chengcheng Wang, Zixiu Liu, Xunhong Liu, Lisi Zou, Jingjing Shi, Shuyu Chen, Jiali Chen, Mengxia Tan

**Affiliations:** 1 College of Pharmacy, Nanjing University of Chinese Medicine, Nanjing 210023, China; cuihuachen2013@163.com (C.C.); ccw199192@163.com (C.W.); liuzixiu3221@126.com (Z.L.); zlstcm@126.com (L.Z.); shijingjingquiet@163.com (J.S.); 18305172513@163.com (S.C.); 18994986833@163.com (J.C.); 18816250751@163.com (M.T.); 2 Collaborative Innovation Center of Chinese Medicinal Resources Industrialization, Nanjing 210023, China; 3 National and Local Collaborative Engineering Center of Chinese Medicinal Resources Industrialization and Formulae Innovative Medicine, Nanjing 210023, China

**Keywords:** Apocyni Veneti Folium, salt stress, multiple bioactive constituents, physiological changes, multivariate statistical analysis

## Abstract

As one of the major abiotic stresses, salinity stress may affect the physiology and biochemical components of *Apocynum venetum* L. To systematically evaluate the quality of Apocyni Veneti Folium (AVF) from the perspective of physiological and the wide variety of bioactive components response to various concentrations of salt stress, this experiment was arranged on the basis of ultra-fast liquid chromatography tandem triple quadrupole mass spectrometry (UFLC-QTRAP-MS/MS) technology and multivariate statistical analysis. Physiological characteristics of photosynthetic pigments, osmotic homeostasis, lipid peroxidation product, and antioxidative enzymes were introduced to investigate the salt tolerance mechanism of AVF under salinity treatments of four concentrations (0, 100, 200, and 300 mM NaCl, respectively). Furthermore, a total of 43 bioactive constituents, including 14 amino acids, nine nucleosides, six organic acids, and 14 flavonoids were quantified in AVF under salt stress. In addition, multivariate statistical analysis, including hierarchical clustering analysis, principal component analysis (PCA), and gray relational analysis (GRA) was employed to systematically cluster, distinguish, and evaluate the samples, respectively. Compared with the control, the results demonstrated that 200 mM and 100 mM salt stress contributed to maintain high quality of photosynthesis, osmotic balance, antioxidant enzyme activity, and the accumulation of metabolites, except for total organic acids, and the quality of AVF obtained by these two groups was better than others; however, under severe stress, the accumulation of the oxidative damage and the reduction of metabolite caused by inefficiently scavenging reactive oxygen species (ROS) lead to lower quality. In summary, the proposed method may provide integrated information for the quality evaluation of AVF and other salt-tolerant Chinese medicines.

## 1. Introduction

As a major abiotic factors constraint on agriculture, salinity affects about 20% of the cultivated lands in the world and nearly 50% of all irrigated lands [1,2]. In China, about 34.6 million hectares lands are suffering from salinity interference. The medicinal plants among the major and vital groups of crops that exert a significant role in disease prevention and treatment are also being threatened by this constraint [3].

The effects of salt stress on plant growth are mainly revealed in ion toxicity, osmotic stress, and secondary oxidative stress. Plants subjected to salt stress form a series of physiological and molecular mechanisms that respond to salt stress, including ion transport and distribution to maintain ion balance [4,5], osmotic adjustment substances, and metabolites formation to maintain osmotic balance, antioxidant enzyme accumulation, and activity enhancement to resist oxidative stress, signal transduction factors, and salt stress-related genes regulation [6,7,8,9,10].

Apocyni Veneti Folium (AVF), Luobumaye in Chinese, is the dried leaf of *Apocynum venetum* L. (Apocynaceae) [11]. For centuries, AVF has been used to treat cardiac disease, hypertension, nephritis, and neurasthenia, and processed into tea due to its health benefits. Documentaries demonstrated that AVF has functions of antihypertension, antidepression, hepatoprotection, antianxiety, antioxidation, and diuresis [12,13]. It is well established that metabolites of medicinal plants, as sources of natural antioxidant and immune enhancer, are involved in the treatment of human diseases and health disorders [14]. However, their synthesis and accumulation depend on the growing conditions and are vitalized under abiotic stresses [5]. Therefore, a systematic quality assessment method is required for the quality control of herbal medicines.

For the assessment of contents of bioactive components in AVF, many analytical methods have been established by using high performance liquid chromatography (HPLC) coupled to an UV detector [15] or high performance capillary electrophoresis method with diode array detection (HPCE-DAD) [16]. However, these methods have been limited to the quantification of only a few flavones. Additionally, ion trap-time-of-flight (IT-TOF) MS system has been applied to sensitively detect phenolic acids and flavonoids in AVF [17], but not simultaneously detect a variety of bioactive compounds. Recently, ultra-fast liquid chromatography tandem triple quadrupole mass spectrometry (UFLC-QTRAP-MS/MS) is useful for the qualitative and quantitative analysis of bioactive compounds with the advantages of great separation efficiency, high peak capacity, and high sensitivity [18,19] and has emerged as a good tool for the sensitive and selective analysis of various constituents [20].

*Apocynum venetum* L., as a medicinal halophytic plant, is able to grow in most regions of China, but widely distributed among saline-alkali wasteland, desert edge, and the Gobi desert. According to the Chinese Pharmacopoeia (2015), hyperoside is used as a basis for assessing the quality of AVF and its content should be no less than 0.3% [13]. However, documents have shown that the content of particular class of components is not efficient to evaluate AVF, considering its growing environment [17,21], and there is no published comparative study reported for the quantification of bioactive constituents combined with physiological changes simultaneously.

Choosing of optimum environmental condition to elevate the metabolites has been reported on medicinal plants [22]. In this study, the effects of salt stress on the physiological characteristics and multiple bioactive constituents of AVF were studied, and quantitative results were then further interpreted by multivariate statistical analysis to evaluate its quality. Our investigation may provide a theoretical basis for the quality evaluation of AVF in respect of the increasing in metabolites under superior salt stressed condition and the mechanism of salt tolerance.

## 2. Results

### 2.1. Physiological Changes Affected by NaCl

#### 2.1.1. Effects of Salt Stress on Photosynthetic Pigments

Salt-treated plant did not show significant change from the perspective of plant phenotypes even at the end of the 40 days experiment (Figure S1), as compared to the control. Chlorophyll a, total chlorophyll, chlorophyll a/b, and carotenoids were significantly increased in the presence of 100 and 200 mM salt, but the changes were not significant under severe stress (Table 1). However, chlorophyll b had little change compared to the control throughout the salt stressed experiments with value ranging from 0.43 to 0.55 mg g^−1^ FW.

#### 2.1.2. Effects of Salt Stress on Osmolytes and Lipid Peroxidation

**Figure 1:**
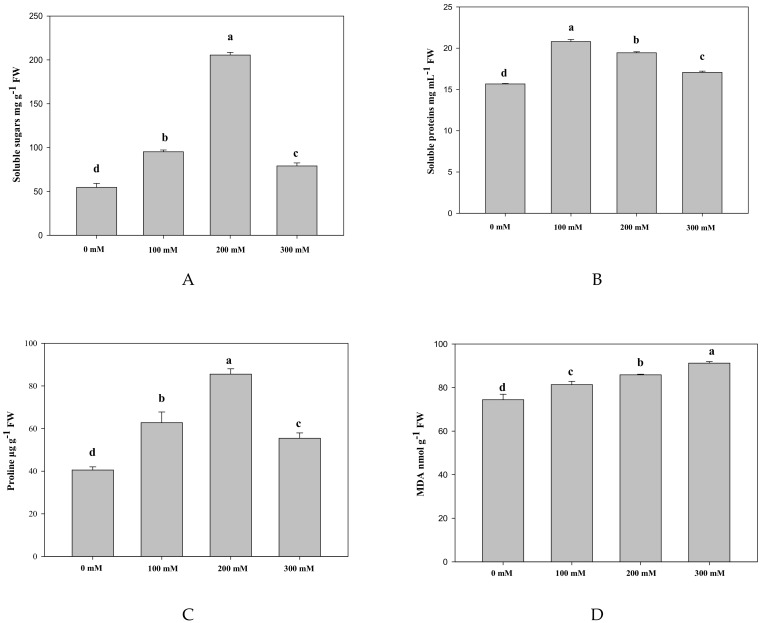
Effects of different concentrations of NaCl on soluble sugars (**A**), soluble proteins (**B**), proline (**C**), and MDA (**D**). Bars are expressed as the mean ± SD (*n* = 3). Bars carrying different letters are significantly different at *p* < 0.05.

Compared to the control, the content of soluble sugars, proline and soluble proteins under salt treatment showed significant changes, which increased first and then decreased throughout the experiment (Figure 1). It is worth noting that the first two increased about 3.76 and 2.11-fold, respectively, and the osmolytes were affected significantly under 200 mM NaCl treatment. The accumulation of malondiadehyde (MDA) was significantly increased with the elevated salt treatments ranging from 74.42 to 91.21 nmol g^−1^ FW (Table S1).

#### 2.1.3. Effects of Salt Stress on Antioxidant Enzyme and Ascorbic Acid

**Figure 2:**
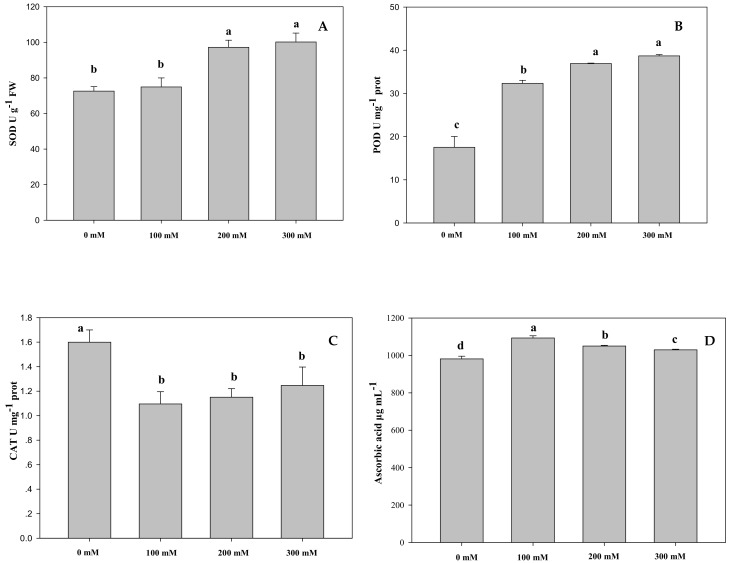
Effects of different NaCl concentrations on the activities of superoxide dismutase (**A**), peroxidase (**B**) catalase (**C**), and ascorbic acid contents (**D**) in Apocyni Veneti Folium (AVF). Bars are expressed as the mean ± SD (*n* = 3). Bars carrying different letters are significantly different at *p* < 0.05 among NaCl treatments.

It can be observed from Figure 2 that the activity of superoxide dismutase (SOD) was significantly increased under moderate stress and severe stress with respect to the control. Peroxidase (POD) activity was noticeably enhanced, specifically under severe stress, increased to 38.66 U mg^−1^ prot, about 2.2-fold compared with the control (Table S1). However, catalase (CAT) activity in salt-treated AVF was significantly declined compared to the control, but it was elevated with the increasing salt concentrations from 100 to 300 mM (Table S1). Significant change was shown between any two groups of ascorbic acid ranging from 980.9 to 1095 μg mL^−1^, but it was moderately higher in the salt-treated groups than in the control.

### 2.2. Determination of Multiple Bioactive Components

#### 2.2.1. Optimization of Sample Preparation and UFLC-QTRAP-MS/MS Conditions

In order to obtain the optimal extraction efficiency, extraction methods were optimized. Relatively speaking, ultrasonic extraction of samples with a ratio of water volume (mL) to sample weight (g) in accordance with 100:1 for 45 min under 30 °C was the appropriate condition. Then, four kinds of standard compounds with low and high contents, uracil, phenylalanine, neochlorogenic acid, and hyperoside were used to optimize the UFLC-QTRAP-MS/MS conditions. By using the UFLC system with a XBridge^®^ C_18_ column (100 mm × 4.6 mm, 3.5 μm) in the case of the mobile phase of 0.1% formic acid in water—0.1% formic acid in acetonitrile, the flow rate of 0.8 mL min^−1^, and the column temperature of 30 °C; the analytes were well separated.

MRM (multiple reaction monitoring) technology mainly targeted selection of data for mass spectrometry signal acquisition, recorded signal of the regular ion pairs, and removed the interference ion signal. Only the MS/MS^2^ ions selected for mass spectrometry acquisition, in order to achieve more specific, sensitive and accurate analysis of the target molecules [20]. Representative extracted ion chromatograms of 43 analytes in the MRM mode were presented in Figure S2, the detailed information about MS/MS condition for each analyte was listed in Table S2, and the characteristic total ion chromatograms (TIC) was displayed in Figure S3.

#### 2.2.2. Method Validation

Quantitative analysis was performed using the UFLC-QTRAP-MS/MS technique. Calibration curves were constructed by injecting each analyte three times and over six suitable concentrations into UFLC-QTRAP-MS/MS system. As listed in Table S3, the limits of detection (LODs) and limits of quantitation (LOQs) were measured in the range of 0.91–6.15 ng mL^−1^ and 3.03–20.5 ng mL^−1^, respectively. Each RSD was less than 5%, and the recovery ranged from 95.19 to 103.91%.

#### 2.2.3. Sample Determination

**Figure 3:**
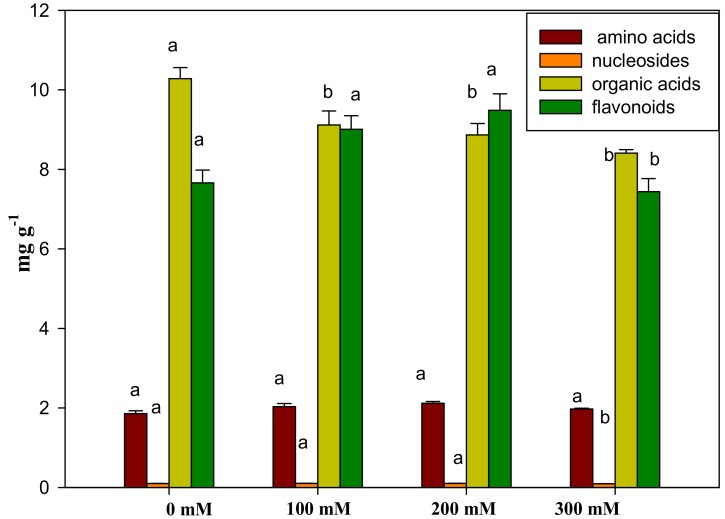
The accumulation of four kinds of constituents in AVF under salt tolerance. Bars are expressed as the mean ± SD (*n* = 3). Bars carrying different letters are significantly different at *p* < 0.05 among NaCl treatments.

Compared with the control, there was no significant change in asparagine, a higher content of amino acids in AVF, except for 200 mM of salinity stressed group (Table S4). The content of glutamine, proline, and glutamic acid changed notably between any two groups, while the change in the total amino acids was non-significant (Figure 3). Little change was detected on the total nucleosides except for the 300 mM NaCl stressed group. By comparison with the control, the accumulation of inosine and thymidine were considerably reduced. The content of neochlorogenic acid and the total organic acids under salinity stress was significantly reduced, but the change in caffeic acid was different. Compared to the control, kaempferol 3-*O*-rutinoside, gallocatechin, and epigallocatechin were significantly increased under salt stress, and significant differences were shown from each other. The accumulations of hyperoside, isoquercitrin, astragalin, trifolin, and total flavonoids were changed similarly, increasing first and then decreasing with the increasing salt concentrations.

#### 2.2.4. Multivariate Statistical Analysis of Samples

**Figure 4:**
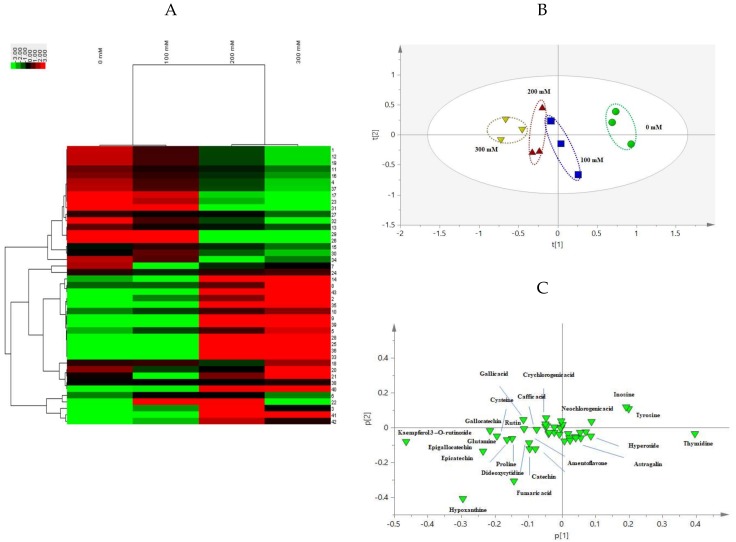
Multivariate statistical analysis of AVF under different salt treatments. Heat map derived from hierarchical clustering analysis (**A**), principal component analysis (PCA) scores plot (**B**), and PCA loading plot (**C**) of AVF.

A heat map derived from hierarchical clustering analysis intuitively displayed the changes of the accumulation of 43 bioactive components under salinity stress (Figure 4A), and on the other hand, the clustering of samples. In detail, 0 and 100 mM salt treated samples, and 200 and 300 mM salt treated ones were clustered separately and then gathered together. Principal component analysis (PCA) scores plot (Figure 4B) exhibited a statistical distinction based on 43 compositions under salinity stress with R2X [1] and R2X [2] accounted for 47.0% and 16.6% of the total variance, respectively [11]. In the PCA loading plot, chemical markers possessing large loading values of ions, such askaempferol 3-*O*-rutinoside, hypoxanthine, and thymidine strongly contribute to sample classification. Additionally, gray relational analysis (GRA) is part of the grey system theory and is suitable for solving problems with complicated interrelationships between multiple factors and variables. It provides a reliable guarantee for the quality evaluation of traditional Chinese medicines. The relative correlation degree (*r_i_*) derived from GRA is proportional to the sample quality. Thus, the quality order of AVF under different NaCl treatments was: 200 mM salinity stressed group > 100 mM salinity stressed group > 300 mM salinity stressed group > control, and the corresponding values of *r_i_* were 0.6363, 0.5253, 0.4827, and 0.3984, respectively. These directly revealed that the accumulations of metabolites were affected under saline condition in AVF (Table 2).

## 3. Discussion

Plants exposed to salt stress undergo physiological and biochemical adaptations to help maintain protoplasmic viability in response to salinity. It has been reported that salt stress can cause oxidative stress through increased reactive oxygen species (ROS). Though ROS molecules are the by-products of vital metabolisms, the built-in antioxidant system maintains the ROS under the controlled level. Temporal and spatial-localization of ROS is vital for the regulation of signaling mechanisms [23]. Highly-accumulated ROS, generated as a result of the decreased gas exchange processes and impairment in protective mechanisms, could damage the cellular components, such as lipids, proteins, and nucleic acids [24]. The increased salinity affects primary carbon metabolism, plant growth, and development by ion toxicity, and induces nutritional deficiency, water deficits, and oxidative stress [5,25]. Moreover, it modulates the levels of secondary metabolites, which are physiologically important particularly under stress tolerance [26]. To mitigate ROS-mediated oxidative damage, plants have developed a complex antioxidant defense system that includes osmotic homeostasis, antioxidant enzymes, and metabolites [27,28,29].

Photosynthesis is involved in the energy metabolism of all the plant systems. When higher plants suffer from salt stress, a growth disorder normally occurs, such as membrane damage and toxic compound accumulation. It can lead to a reduction of the chlorophyll content, disintegration of chloroplast membranes, disruption of photosystem biochemical reactions, and the reduction of photosynthetic activity [30]. The decrease in the chlorophyll content under environmental stress could be attributed to the enhancement of chlorophyll degradation [31]. Carotenoids are accessory light harvesting pigments, preventing the photosynthetic pigments from photo-damage, stabilizing the phospholipids, and scavenging various ROS generated during stressful salinity.

Compared with the control, the content of pigments shown in this study was increased under low and moderated stress, but decreased under severe stress. It indicates that salt stress at specific concentrations might promote the photosynthesis of AVF and severe stress might have a certain inhibitory effect, probably due to the impact of salt on disturbing photosynthesis process, photosynthetic enzymes, chlorophylls, and carotenoids, respectively [5]. The change of chlorophyll a/b reflects the photosynthetic activity of the leaves, and a reasonable value can prevent the excessive light energy in the leaves from inducing the generation of free radicals and photo oxidation of pigment molecules. In summary, it indicated a full utilization of light energy and enhancement of metabolic activity of AVF exposed to low concentrations of NaCl.

The accumulation of osmotic adjustments is one of salt-tolerant mechanisms. It may increase cellular concentrations, help maintain ion homeostasis and water relations, alleviate the negative effects of high ion concentrations on the enzymes and proteins under stressed conditions [32]. Soluble sugars are involved in biosynthetic process and can balance the osmotic strength of cytosol with that of vacuole [33]. The fluctuation in this study under salt stress might be caused by the changes of CO_2_ assimilation, source-sink carbon partitioning, and/ or the activity of related enzymes [34]. Soluble proteins in AVF clearly increased depending on the rising of NaCl, and then significantly reduced, indicating that cell turgor maintaining and water acquisition regulation perhaps was affected by salt stress [35,36]. The results were consistent with the literature reported on *Salvia miltiorrhiza* [37]. The increased osmolytes in AVF might be responsible for maintaining homeostasis under low and moderate salt stress, but under severe salt stress, it was notably affected, possibly due to the inability to effectively keep osmotic balance.

Proline accumulation is one of the adaptations of plants to salinity. It has a wide range of biological functions in plants, such as scavenging free-radical by quenching of singlet oxygen, protecting macromolecules against denaturation [38,39,40], reducing the acidity in the cell, and helping rapid growth after stress [41]. In this study, proline alleviated NaCl stressed induction, but it was significantly reduced caused by osmotic tolerance due to the severe salt stress [42]. 

Under severe salinity stress, loss of the membrane integrity and stability is a common symptom developed in plants [43] due to excessively formation of free radicals and lipid peroxidation. As one of the lipid peroxidation products, MDA plays an important role in modifying core proteins and in many stressed plants, and it is considered as a useful oxidative marker to indicate the chloroplast lipid peroxidation [44]. A significant increase in the MDA content with salt stress elevated in our study might indicate the oxidative degradation of chloroplast membranes [33,45].

SOD, CAT, and POD, which are involved in antioxidation processes, protect plants from oxidative damage caused by abiotic stresses [46]. SOD catalyzes the dismutation of O_2_^−^ into O_2_ and H_2_O_2_, whereas CAT dis-mutates mostly photorespiratory/respiratory H_2_O_2_ into H_2_O and O_2_, and POD is responsible for the removal of H_2_O_2_ by oxidation of co-substrate, such as phenolic compounds [47]. Integral coordination of antioxidant enzymes could be vital for the redox homeostasis mechanism under the oxidative stress, as previously reported in wheat [48] and barley [49]. In the present study, elevated NaCl increased the activities of SOD and POD, but not CAT. Furthermore, POD might be efficient in clearing the excess H_2_O_2_ with significantly increased activity and slightly increased SOD activity but significantly decreased in CAT activity appeared under 50 mM NaCl stress. Besides H_2_O_2_ stress, salt stress might simultaneously enhance superoxide production in cells [50].

As a non-enzymatic free radical scavenger and a key substance in the network of antioxidants, ascorbic acid has been shown to play multiple roles in plant growth. It has also been seen in regulating the normal reactive oxygen species in plant cells together with other small molecules [51,52]. A significant increase was shown in ascorbic acid to protect the body from endogenous damage of oxygen free radicals and then a decrease with the increasing NaCl concentrations, which might be due to the consumption as an enzyme-catalyzed substrate for scavenging ROS.

The increased level of free amino acids in the cell cytoplasm plays an important role in osmotic adjustments, which are also involved in the stability and integrity of cellular membranes in saline environment [53]. The amino acid content was found increased in *Aloe vera* during salinity stress [54]. In this study, the elevated level of amino acids, such as glutamic acid, cysteine, and proline helped provide osmotic protection for AVF. Nucleosides and their derivatives have significant physiological functions. Higher salinity had been reported to induce changes in protein structure, increase in cytoplasmic RNAase activity, leading to decrease in DNA synthesis and creating many cellular menaces to activity required for development processes in plants [55]. Under adverse conditions, transcription factors associated with stress resistance can regulate the simultaneous expression of multiple stress-tolerant genes and the transmission of stress signals [56]. Lower content of nucleosides was shown under severe stress due to the salt stress.

Abiotic stress promotes the synthesis of various secondary metabolites possessing antioxidant activity. Organic acids and flavonoids are ubiquitous in plants and are generally accumulated in response to salinity stress [57]. As mentioned above, phenolic metabolites can cooperate with POD in H_2_O_2_ scavenging. The phenylpropanoid pathway is the main metabolic route for the synthesis of phenolics and flavonoids [58]. The accumulation of organic acids may vary in different plant species in response to salinity tolerance. In previous reports, they were observed increased in buckwheat sprout [59], but decreased in baby Romaine lettuce [60]. In this experiment, they were significantly depressed under salinity conditions, possibly because of the consumption of scavenging or detoxifying excess free radicals [61].

Flavonoids have a wide array of physiological functions in plants, e.g., involvement in UV filtration and symbiotic nitrogen fixation, and acting as chemical signal messengers for initiating plant-microbe symbiotic associations [62,63], and they also contribute significantly for human by virtue of antioxidative, antiviruses, antiangiogenic, and neuropharmacological effects [64,65]. Flavonoid biosynthesis can be stimulated by the variation of the cellular redox homeostasis and lipid peroxidation of membranes of the plant cell [33,66]. In our study, the increase in flavonoid content under low and moderate might be associated with the increases in chlorophyll, and this enhanced synthesis of secondary metabolites under stressful conditions was believed to protect the cellular structures from oxidative damage and osmotic stress [67]. However, it decreases under severe salt stress, which might be due to the oxidative damage caused by the imbalance between antioxidants formation and ROS scavenging.

According to the results of hierarchical clustering analysis showed in the heat map, the control and low concentrations of salt stressed AVF, and the moderate and high concentrations of salt stressed were aggregated together orderly. This was consistent with PCA results that AVF samples exposed to salt stress (0, 100, 200, and 300 mM, respectively) were sequentially distributed and could be distinguished from each other from the positive to the negative axis of PC1. Moreover, chemical markers in the PCA loading plot provided the possibility of sample differentiation. In all, the results of multivariate statistical analysis indicated that it was not only provided relevant basis for the classification of various salt treatment of AVF, but also comprehensively evaluated the quality of it, that is, moderate salt treated samples were superior to others, and salt treated ones were better than the control.

Generally speaking, in plants under salt stress, photosynthesis, osmotic balance, and metabolic processes are deeply affected [5]. Impairment in the photosynthetic process leads to the higher lipid peroxidation and excessive accumulation of reactive oxygen species (ROS) [24]. The productions of ROS are much higher than its detoxification in abiotic stress conditions. Osmolytes play key roles in maintaining normal osmotic potential, and antioxidant systems of enzymes and metabolites protect plants from oxidative damage and efficiently retain more tolerance against abiotic stresses. In the subsequent experiments, we will use multi-omics approaches to comprehensively interpret the effects of salt stress on the quality of AVF and the salt tolerance mechanism at the transcription, protein, and metabolic levels [68].

## 4. Materials and Methods

### 4.1. Plant Materials and Salinity Treatments

The experimental samples of AVF were obtained by the following steps. Firstly, the site for salt stress test was selected in Medicinal Botanical Garden of Nanjing University of Traditional Chinese Medicine (latitude 118°57′1′′, East longitude 32°6′5′′). The experiment was carried out in the shelter covered by a transparent film that blocked rainwater, while other conditions were similar to the open-air environment. Secondly, the materials and methods of salt stress were given as follow: The botanical origins of the materials were identified by Professor Xunhong Liu (Department for Authentication of Chinese Medicines, Nanjing University of Chinese Medicine); the main root of *Apocynum Venetum* L., two years old from the same plant, and the number of bud and head close to each other, was excavated from the garden in December 2016, and then planted in pots (50 cm height, 34 cm of top diameter, and 26 cm of bottom diameter). Each pot was filled with 25 kg of dry soil and 3 roots, and was placed in the open air before the salt-treated experiments.

Salt stress tests had been conducted since 20 May 2017 when *Apocynum venetum* L. was in normal growth (about 30 cm height). Four levels of salt treatment concentrations, 0 (control, watering), 100 (low stress), 200 (moderate stress), and 300 mM (severe stress) NaCl treatments were designed with 3 replicates at each concentration level and 3 pots per replicate. According to the previous research, by calculating the amount of water, the final determination of the solution per pot was 2 L. In order to prevent osmotic shock, salt concentrations increased gradually by 50 mM NaCl every four days until the designated concentration was reached and lasted 6 times. The photograph of changes of plant phenotypes upon treatment of different concentrations of NaCl was seen in Figure S1. At last, experimental samples were harvested on 30 June 2017. The collected samples of four groups were immediately frozen at −80 °C for subsequent experiments, some for physiological experiments, some for quantitative analysis, and the rest for the voucher specimens deposited at the Herbarium in School of Pharmacy, Nanjing University of Chinese Medicine.

### 4.2. Physiological Experiment

#### 4.2.1. Extraction and Assay of Pigment

Four groups of fresh AVF samples (0.2 g) under salt stress were homogenized with ethanol (95%, *v*/*v*), filtered, and made up to 2 mL, respectively. Photosynthetic pigments (chlorophyll a, b, total chlorophyll and carotenoids) concentrations were calculated from the absorbance of extract at 665, 649 and 470 nm using the formula [37,69], given as follow: Chlorophyll a (mg g^−1^ FW) = (13.95 × A665 − 6.88 × A649) × 2/(1000 × 0.2); Chlorophyll b (mg g^−1^ FW) = (24.96 × A649 − 7.32 × A665) × 2/(1000 × 0.2); Carotenoids (mg g^−1^ FW) = ((1000 × A470) − (2.05 × Chl a) − (114.8 × Chl b)) × 2/(245 × 1000 × 0.2)

#### 4.2.2. Osmolytes and MDA Assay

Osmolytes, including soluble proteins, soluble sugars and proline, were assayed in this study to measure the salt tolerance of AVF. The content of soluble proteins was determined according to the ultraviolet absorption method. 0.5 g of fresh sample of AVF under salt stress was homogenized and extracted by 8 mL of PBS (phosphate buffer saline, 0.1 mM Na_2_HPO_4_ and NaH_2_PO_4_, pH 7.4), respectively. After centrifugation, 1 μL of the supernatant was subjected to detect using UV-Vis spectrophotometer (DENOVIX DS-11, Wilmington, DE, USA), which can directly display the concentration of protein in the solution by detecting the absorbance of the solution at 260 nm and 280 nm with 1 μL of PBS as a control. The content of soluble sugars and proline was quantified by colorimetric method. To measure the content of soluble sugars, 0.5 g of fresh leaves was homogenized with 4.5 mL of PBS in an ice water bath and centrifuge the homogenate at 3500 rpm for 10 min. 0.5 mL of extract was mixed with 3 mL of anthrone solution (75 mg anthrone in 50 mL of 72% sulphuric acid (*w*/*w*)), and was immediately placed in a boiling water bath for 10 min. The light absorption was estimated at 620 nm. The content of soluble sugars was determined by using glucose as a standard and expressed as mg g^−1^ FW. With regard to the free proline assay, the procedure was as follows: 0.5 g of harvested leaf fragments were extracted with 4.5 mL of aqueous sulfosalicylic acid (3%, *w*/*v*) in boiled water for 10 min and then centrifuged at 3500 rpm for 10 min. After that, 2 mL of glacial acetic acid and 4 mL of acidninhydrin agent (1.25 g acidninhydrin in 30 mL glacial acetic acid and 20 mL of 6 mol L^−1^ H_3_PO_4_) were added to the homogenate in a test tube. The mixture was incubated in boiling water for 30 min, and then the test tube was placed in the cold water to terminate the reaction. Each test tube was added to 4 mL of toluene and vortexed for 30 s. The supernatant was taken and centrifuged at 3000 rpm for 5 min. Proline content was quantified by using the Bate’s method at 520 nm [69,70].

Lipid peroxidation was measured in terms of MDA by thiobarbituric acid (TBA) method [69,71]. Fresh leaf (0.5 g) fragment were homogenized with 5 mL of trichloroacetic acid (3%, *m*/*v*), and then centrifuged at 3000 rpm for 10 min. Took 2.0 mL aliquot of the supernatant to the test tube and added 2.0 mL of thiobarbituric acid (0.67%, *m*/*v*). The mixture was heated in boiling water for 30 min and then quickly cooled in an ice bath. After centrifugation at 3000 rpm for 10 min, the absorbance of the samples was recorded at 530 nm.

#### 4.2.3. Enzyme Activities and Ascorbic Acid Assay

To determine the antioxidant enzyme activities, 0.5 g of fresh AVF under the stress of different salt concentrations were homogenized with 4.5 mL of PBS in an ice water bath and centrifuged at 3500 rpm for 10 min. The supernatant was collected to determine the antioxidant enzyme activities. SOD activity was assayed by hydroxylamine method, CAT activity was determined by ammonium molybdate method, and POD activity was measured according to the colorimetric method [69,70]. As for the determination of ascorbic acid, it was assayed based on the oxidation of ascorbic acid by iron (III) in the presence of 1,10-phenanthroline with subsequent formation of ferroin and a suitable anion associate according to the Zenki et al. method [72]. All of them were tested by assay kits bought from Nanjing Jiancheng Bioengineering Institute (Nanjing, China). UV-visible absorptions were measured by multi-mode microplate reader (SpectraMax M5, San Jose, CA, USA) and the detection amount of the reaction solution was 200 μL.

### 4.3. Multiple Bioactive Constituents Assay

#### 4.3.1. Chemicals and Reagents

Ultrapure water was prepared using a Milli-Q purifying system (Millipore, Bedford, MA, USA). Methanol and acetonitrile of HPLC grade were purchased from Merck (Damstadt, Germany). Standard compounds of histidine (1), arginine (3), cysteine (4), asparagine (5), serine (6), lysine (7), glutamine (8), proline (9), cytidine (10), hypoxanthine (11), deoxycytidine (12), uridine (13), tyrosine (14), guanine (15), guanosine(16), inosine(17), deoxyguanosine (19), isoleucine (20), leucine (21), thymidine (23), phenylalanine (24), tryptophan (27), epicatechin (33), rutin (34), hyperoside (35), and quercitrin (37) were purchased from Shanghai Yuanye Biotechnology (Shanghai, China); glutamic acid (2), gallic acid (22) and apigenin (43) were obtained from Chinese National Institute of Control of Pharmaceutical and Biological Products (Beijing, China); fumaric acid (18), gallocatechin (25), epigallocatechin (28), cryptochlorogenic acid (31), kaempferol 3-*O*-rutinoside (39) and amentoflavone (42) were acquired from Chengdu Chroma Biotechnology (Chengdu, China); neochlorogenic acid (26), chlorogenic acid (29), catechin (30), caffeic acid (32), isoquercitrin (36), avicularin (38), trifolin (40), and astragalin (41) were bought from Baoji Chenguang Biotechnology Co., LTD. (Baoji, China) with the purity greater than 98% and their structures were presented in Figure S4.

#### 4.3.2. Sample Preparation

Four groups of fresh AVF harvested under salt treatments were naturally dried, and then powdered and passed through a 60-mesh sieve. 0.3 g of sample was weighed accurately and ultrasonically extracted with 30 mL of water for 45 min, supplemented with water to compensate for the lost weight, and centrifuged at 12000 rpm for 15 min [11,73]. The supernatant was stored at 4 °C and filtered through a 0.22 μm membrane (Jinteng laboratory equipment Co., Ltd., Tianjin, China) before being subjected to UFLC-MS/MS analysis.

#### 4.3.3. Chromatographic and Mass Spectrometric Conditions

The mobile phase of AB Sciex QTRAP^®^ 4500 UFLC-MS/MS spectrometry consisted of water containing 0.1% formic acid (*v*/*v*, A) and acetonitrile containing 0.1% formic acid (*v*/*v*, B). The analytes were eluted using a linear gradient program: 1–3 min, 5% B; 3–6 min, 5–15% B; 6–15 min, 15–20% B; 15–17 min, 20–70% B, 17–17.5 min, 70–5% B, and 17.5–23 min, 5% B. The flow rate was 0.80 mL/min. The column temperature was 30 °C. The injection volume was 1 μL. According to our previous reports [11], the standard solution of each analyte was injected separately into the electrospray ionization (ESI) source in the direct infusion mode of MS to acquire the fragmentor voltage and collision energy in both positive and negative modes. Next, ESI source operates in both ion modes using the MRM transition acquiring the spectra and the Analyst 1.6.3 software analyzing data, respectively. In the same ion mode, isomers with the same ion pairs, such as catechin/epicatechin, chlorogenic acid/neochlorogenic acid/cryptochlorogenic acid, gallocatechin/epigallocatechin, hyperoside/isoquercetin, and leucine/isoleucine were separately and injected into UFLC-QTRAP-MS/MS to find the accurate *t*_R_ for identification and quantification. The operating parameters were set as follows: GS1 flow, 65 L min^−1^; GS2 flow, 65 L min^−1^; and CUR flow, 30 L min^−1^; gas temperature, 650 °C; pressure of the nebulizer, 5500 V for the positive ion mode, and −4500 V for the negative ion mode, respectively.

#### 4.3.4. Method Validation and Sample Determination

The standard solution containing 43 reference substances was prepared and diluted with water to appropriate concentrations for the construction of calibration curves. The concentrations of 43 analytes in mixed solution were seen in Table S3. The LODs and LOQs of constituents were measured at signal-to-noise (S/N) ratios of 3 and 10, respectively. Precision of the intra and inter-day was expressed as relative standard deviation (RSD). Repeatability was achieved by six different analytical sample solutions prepared by the same sample, and stability was performed by analyzing the variations at 0, 2, 4, 8, 12, and 24 h, respectively. While the recovery test was performed by adding a known amount of corresponding constituents in triplicate at low, medium, or high levels to 0.5 g of 100 mM NaCl treated samples, respectively. The quantitative determination of the bioactive compounds of AVF under different sat stress was performed under the optimal condition by UFLC-QTRAP-MS/MS.

#### 4.3.5. Multivariate Statistical Analysis

Hierarchical cluster analysis is a method of cluster analysis, which seeks to build a hierarchy of clusters. PCA is a statistical procedure that uses an orthogonal transformation to convert a set of observations of possibly correlated variables (entities each of which takes on various numerical values) into a set of values of linearly uncorrelated variables called principal components [11]. Hierarchical clustering analysis and PCA were introduced to cluster and classify samples based on the content of constituents by Java Treeview 3.0 software and SIMCA-P 13.0 software, respectively. Then, GRA was performed according to the contents of 43 bioactive components by Microsoft Excel 2010 for Window 10 to evaluate the quality of AVF under different concentration of salt stress. Specifically, through the establishment of sample dataset and normalization treatment of raw data, the optimal and the worst reference sequences were conducted. After establishing dimension of the differences between comparing sequences and reference sequences, correlation coefficient and correlation degree were calculated, followed by the weight value of the evaluation samples (*r_i_*).

### 4.4. Data Processing

The mean values of all parameters were taken from the measurements of three replicates with the standard deviation calculated. One-way ANOVA followed by Duncan’s multiple-range test was used to compare the means with the significance level set as 0.05 by SPSS 19.0

## 5. Conclusions

In this study, our aim was to use the changes in physiology and biochemical components as references to study the quality control of AVF response to salt stress. Thus, an efficient analytical method of simultaneous determination of multiple bioactive constituents combined with physiological analysis was established for the quality evaluation based on the multivariate statistical analysis. Investigations into the physiological changes of photosynthetic pigments, osmotic homeostasis, lipid peroxidation, antioxidative enzymes, and ascorbic acid could provide comprehensive insights into the response mechanisms induced by salt stress. Furthermore, a total of 43 bioactive constituents, including amino acids, nucleosides, organic acids, and flavonoids, were successfully identified and quantified in different salinity-treated AVF with the application of UFLC-QTRAP-MS/MS technology. Multivariate statistical analysis was performed for the group classification and quality evaluation. Overall, the quality of AVF subjected to NaCl was superior to the control and AVF treated with 200 mM NaCl had the best quality. In general, this study was conducted to the quality evaluation of AVF concerning the impacts caused by salinity on the physiology and bioactive constituents. The results might provide a valuable reference for the quality assessment of other herbal medicines and the development of salt-tolerant plants in saline soils.

## Figures and Tables

**Table 1:** Effects of different NaCl concentrations on the chlorophyll (Chl) and carotenoids contents of AVF.

Treatments	Pigment Content				
	Chl a (mg g^−1^ FW)	Chl b (mg g^−1^ FW)	total Chl (mg g^−1^ FW)	Chl a/b	Carotenoids (mg g^−1^ FW)
0 mM	2.35 ± 0.11 c	0.49 ± 0.06 ab	2.84 ± 0.05 c	4.77 ± 0.82 c	0.86 ± 0.07 c
100 mM	3.66 ± 0.19 a	0.55 ± 0.04 a	4.21 ± 0.22 a	6.63 ± 0.14 a	1.25 ± 0.01 a
200 mM	3.00 ± 0.20 b	0.48 ± 0.01 ab	3.47 ± 0.20 b	6.30 ± 0.49 ab	1.04 ± 0.02 b
300 mM	2.31 ± 0.12 c	0.43 ± 0.01 b	2.74 ± 0.11 c	5.33 ± 0.40 bc	0.83 ± 0.01 c

Data are the mean ± SD (*n* = 3). Different letters following values in the same column indicate significant difference among salt treatments using Duncan’s multiple-range test at *p* < 0.05.

**Table 2:** Quality sequencing of the tested samples affected by NaCl.

Treatments	*r* *_i_*	Quality-Ranking
0 mM	0.3984	4
100 mM	0.5253	2
200 mM	0.6363	1
300 mM	0.4827	3

## Supplementary Materials

Supplementary materials can be found at http://www.mdpi.com/1422-0067/19/10/3042/s1.
