# Supplementary material for: Variations in Physiology and Multiple Bioactive Constituents under Salt Stress Provide Insight into the Quality Evaluation of Apocyni Veneti Folium
Source: Int J Mol Sci. 2018 Oct 5;19(10):3042. doi: 10.3390/ijms19103042 (PMC6213253; doi:10.3390/ijms19103042)
Supplement: Supplementary file 1 [file ijms-19-03042-s001.pdf]

Supplementary Materials

Variations in Physiology and Multiple Bioactive Constituents under Salt Stress Provide Insight into the Quality Evaluation of Apocyni Veneti Folium

Cuihua Chen [1], Chengcheng Wang [1], Zixiu Liu [1], Xunhong Liu[1,2,3,*], Lisi Zou [1], Jingjing Shi [1], Shuyu Chen [1], Jiali Chen [1] and Mengxia Tan [1]

1 College of pharmacy, Nanjing University of Chinese Medicine, Nanjing 210023, China; cuihuachen2013@163.com (C.C.); ccw199192@163.com (C.W.); liuzixiu3221@126.com (Z.L.); zlstcm@126.com (L.Z.); shijingjingquiet@163.com (J.S); 18305172513@163.com(S.C.); 18994986833@163.com (J.C.); 18816250751@163.com (M.T.)

2 Collaborative Innovation Center of Chinese Medicinal Resources Industrialization, Nanjing 210023, China

3 National and Local Collaborative Engineering Center of Chinese Medicinal Resources Industrialization and Formulae Innovative Medicine, Nanjing 210023, China

* Correspondence: liuxunh1959@163.com; Tel./Fax: +86-25-8581-1524

Figure S1 Morphological changes in AVF after the treatment of different concentrations of NaCl

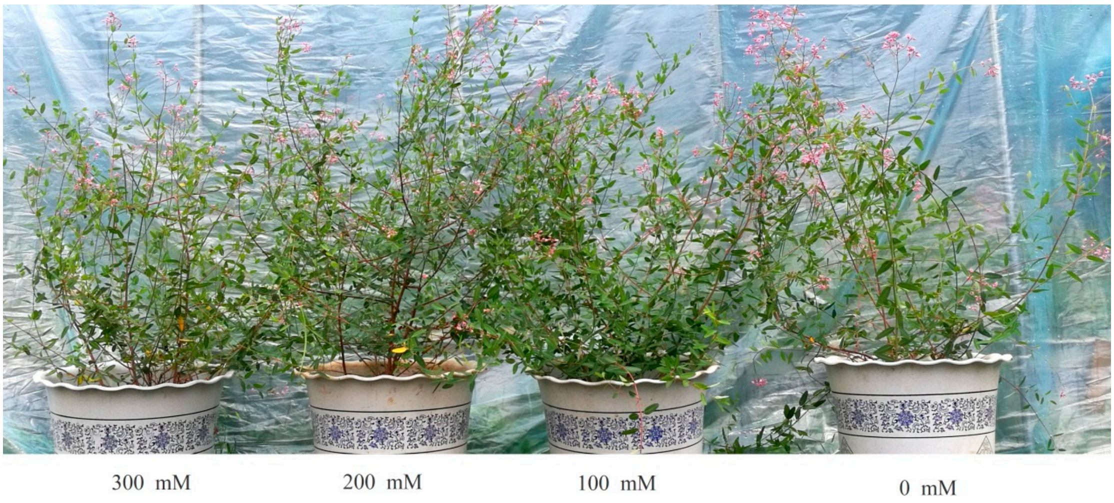

Table S1 Physiological data of AVF under salt stress

Physiological parameters	NaCl treatments			
	0 mM	100 mM	200 mM	300 mM
soluble sugars(mg g^{-1} FW)	54.55± 4.50d	95.08± 2.01b	205.17± 3.01a	79.35± 3.51c
soluble proteins(mg mL $^{-1}$ FW)	15.67± 0.06d	20.80± 0.26a	19.45±0.12b	17.07±0.15c
Proline(μg g^{-1} FW)	40.54± 1.50d	62.77± 5.02b	85.54± 2.50a	55.46± 2.50c
MDA (nmol g^{-1}FW)	74.42± 2.50d	81.33± 1.53c	85.83± 0.29b	91.21± 0.71a
SOD(U g^{-1} FW)	72.56±2.50 b	74.92± 5.05b	97.18± 4.01 a	100.18± 5.01a
CAT (U mg^{-1} prot)	1.60± 0.10 a	1.10± 0.10b	1.15± 0.07 b	1.25± 0.15b
POD (U mg^{-1} prot)	17.52± 2.50c	32.33± 0.70b	36.92±0.10a	38.66± 0.30a
ascorbic acid (μg mL^{-1})	980.9±14.50 d	1095.00±12.12 a	1050.00±4.00 b	1029.67±3.51 c

a, b, c and d Data are the mean ± SD (n = 3). Different letters following values in the same row indicate significant difference among salt treatments using Duncan's multiple-range test at $p < 0.05$.

Figure S2 Representative extracted ion chromatograms of MRM chromatograms of the AVF under salt stress.

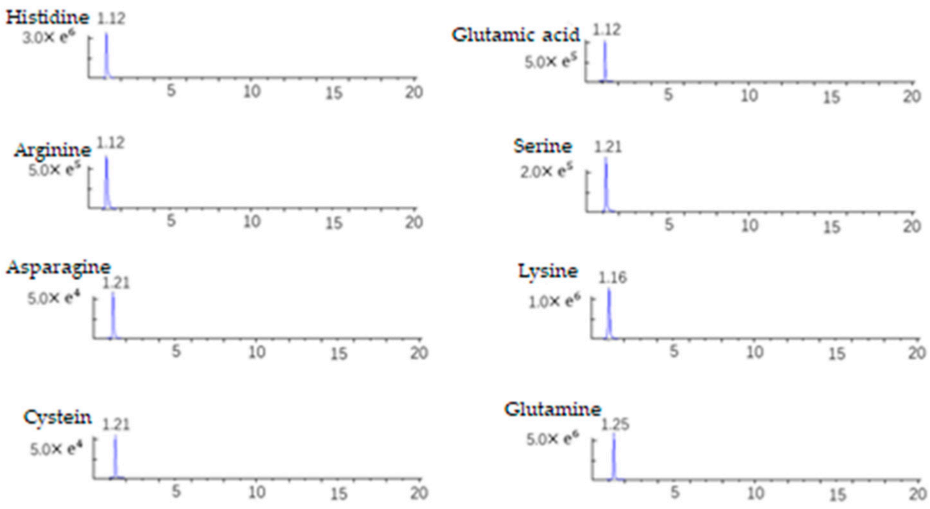

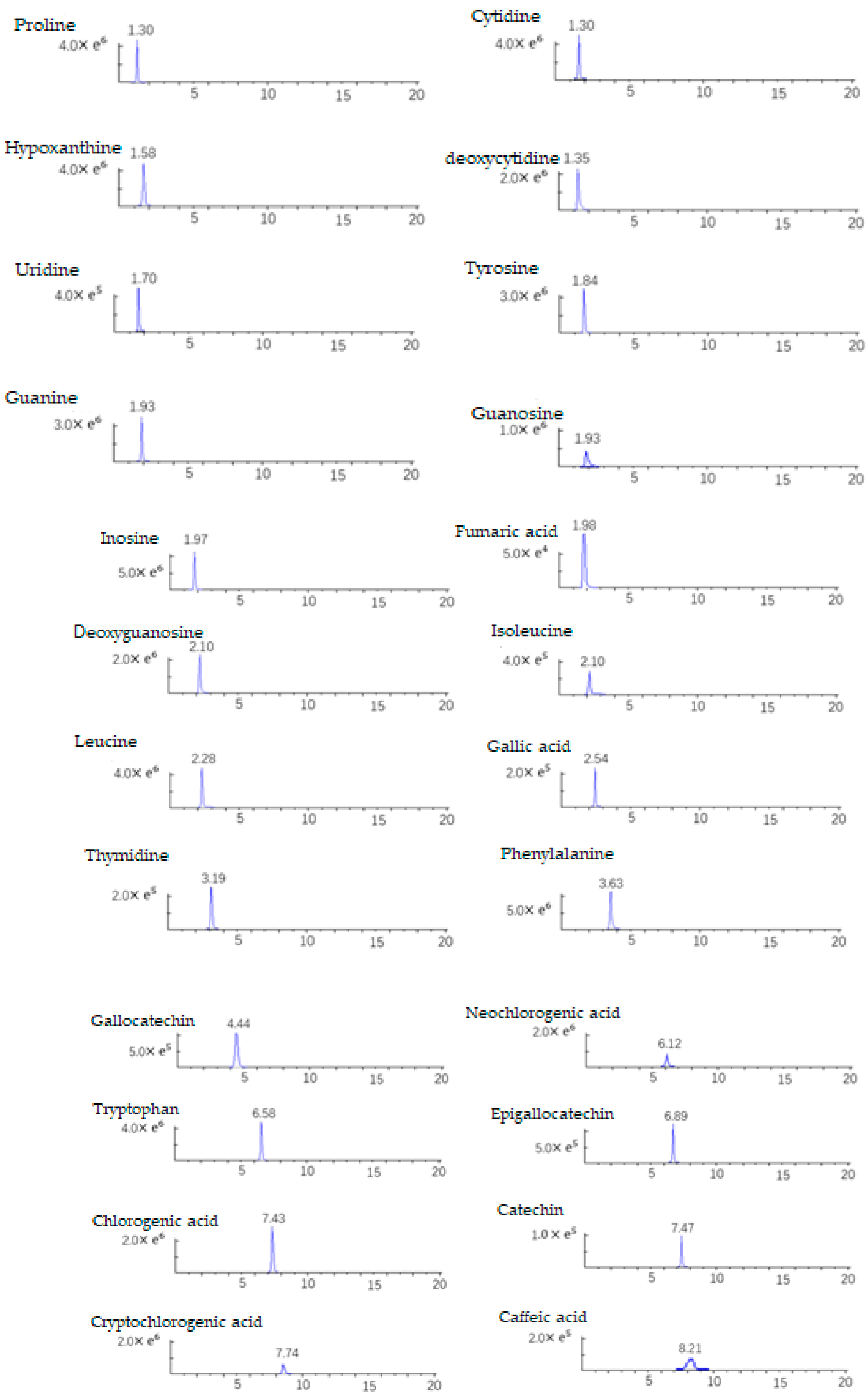
Proline
1.30
4.0× e6
Cytidine
1.30
4.0× e6
Hypoxanthine
1.58
4.0× e6
deoxycytidine
1.35
2.0× e6
Uridine
1.70
4.0× e5
Tyrosine
1.84
3.0× e6
Guanine
1.93
3.0× e6
Guanosine
1.93
1.0× e6
Inosine
1.97
5.0× e6
Fumaric acid
1.98
5.0× e4
Deoxyguanosine
2.10
2.0× e6
Isoleucine
2.10
4.0× e5
Leucine
2.28
4.0× e6
Gallic acid
2.54
2.0× e5
Thymidine
3.19
2.0× e5
Phenylalanine
3.63
5.0× e6
Gallocatechin
4.44
5.0× e5
Neochlorogenic acid
6.12
2.0× e6
Tryptophan
6.58
4.0× e6
Epigallocatechin
6.89
5.0× e5
Chlorogenic acid
7.43
2.0× e6
Catechin
7.47
1.0× e5
Cryptochlorogenic acid
7.74
2.0× e6
Caffeic acid
8.21
2.0× e5
5
10
15
20

Table S2 Parameters for multiple reaction monitoring (MRM) of the 43 target constituents.

NO.	Constituents	t_R (min)	*m*/*z*	Precursor ion	Product ion	Fragmentor voltage (V)	Collision energy (eV)
1	Histidine	1.11	155[M + H]+	156.08	110.03	95	16
2	Glutamic acid	1.12	147.13[M + H]+	147.08	83.92	83	14
3	Arginine	1.13	174.2[M + H]+	175.12	70.02	88	18
4	Cysteine	1.21	122.1529[M + H]+	122.03	75.93	85	17
5	Asparagine	1.22	133.1279[M + H]+	133.06	73.87	65	14
6	Serine	1.23	105.09[M + H]+	106.05	59.99	67	8
7	Lysine	1.23	146.19[M + H]+	147.11	83.91	66	14
8	Glutamine	1.26	147.1579[M + H]+	148.06	83.91	58	14
9	Proline	1.30	115.13[M + H]+	116.07	70.02	68	10
10	Cytidine	1.31	243.22[M + H]+	244.09	112.0	61	10
11	Hypoxanthine	1.34	136.11[M + H]+	137.05	137.05	51	24
12	deoxycytidine	1.39	227.3[M + H]+	228.2	112.05	76	13
13	Uridine	1.70	244.2[M + H]+	244.9	113.0	103	13
14	Tyrosine	1.84	182.1[M + H]+	182.16	136.08	46	17
15	Guanine	1.93	151.12[M + H]+	152.0	135.0	51	25
16	Guanosine	1.95	283.24[M + H]+	284.3	152.0	62	15
17	Inosine	1.97	268.23[M + H]+	269.0	137.07	46	15
18	Fumaric acid	1.99	115.0621[M − H]	114.8	70.8	-50	-10
19	deoxyguanosine	2.07	267.2[M + H]+	268.1	152.1	61	15
20	Isoleucine	2.09	131.18[M + H]+	132.1	86.05	98	10
21	Leucine	2.28	131.18[M + H]+	132.1	86.05	98	10
22	Gallic acid	2.54	169.1121[M − H]−	168.9	125.0	-120	-18
23	Thymidine	3.17	242.23[M + H]+	243.1	127.07	61	13
24	Phenylalanine	3.60	165.19[M + H]+	166.1	120.05	56	14
25	Gallocatechin	4.43	305.262[M − H]−	305.1	125.0	-55	-25
26	Neochlorogenic acid	6.12	353.09[M − H]−	305.01	125.0	-95	-20
27	Tryptophan	6.59	205.2379[M + H]+	205.03	188.1	61	13
28	Epigallocatechin	6.88	305.262[M − H]−	305.1	125.0	-55	-25
29	Chlorogenic acid	7.43	353.09[M − H]−	305.01	125.0	-95	-20
30	Catechin	7.47	289.2621[M − H]−	289.0	244.8	-135	-20
31	Cryptochlorogenic acid	7.74	353.09[M − H]−	305.01	125.0	-95	-20
32	Caffeic acid	8.19	179.1421[M − H]−	178.97	134.6	-125	-20
33	Epicatechin	8.54	289.2621[M − H]−	289.0	244.8	-135	-20
34	Rutin	11.10	609.5[M − H]−	609.06	300.0	-245	-46
35	Hyperoside	11.57	463.37[M − H]−	463.0	300.0	-180	-36
36	Isoquercitrin	11.9	463.3721[M − H]−	463.0	300.0	-180	-36
37	Quercitrin	13.30	447.3681[M − H]−	447.0	301.0	-165	-30
38	Avicularin	13.30	435.3579[M + H]+	435.0	303.0	51	15
39	Kaempferol 3- O-rutinoside	13.32	595.5279[M + H]+	595.0	287.2	36	25
40	Trifolin	13.34	449.3239[M + H]+	449.0	287.0	46	21
41	Astragalin	14.26	447.36[M − H]−	447.1	283.9	-100	-36
42	Amentoflavone	17.80	539.4579[M + H]+	539.0	377.0	251	57
43	Apigenin	18.10	269.2321[M − H]−	268.8	116.9	-129	-40

Figure S3 Total ion chromatogram of AVF under salt stress

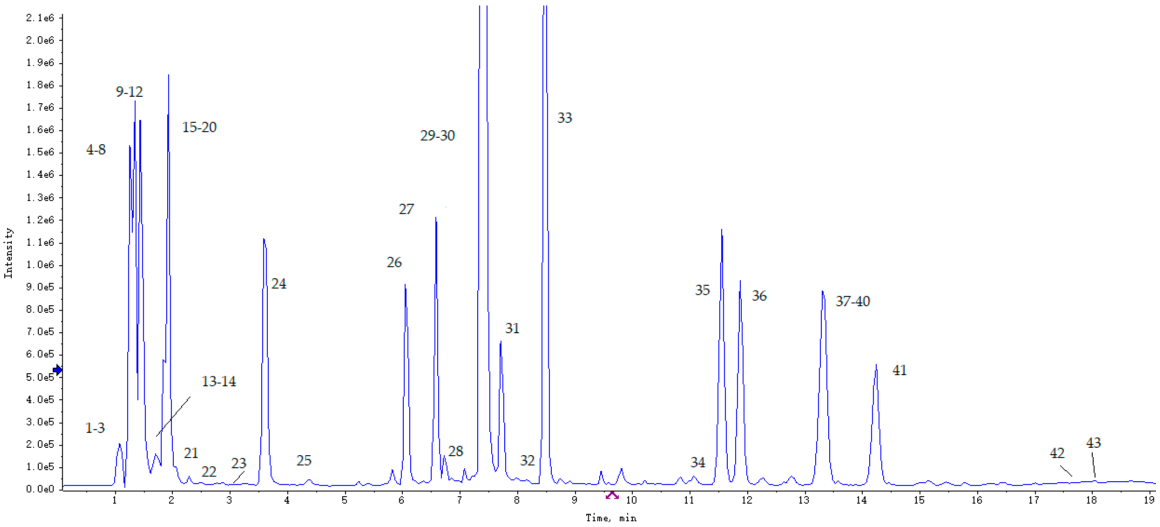

The order of 1-43 was the same as the number in Table S2.

Table S3. Regression equation, correlation coefficient, linear range, limit of detection (LODs), limit of quantitation (LOQs), precision, repeatability, stability, and recovery of investigated constituents.

Analytes	Concentrations ($\mu g\ mL^{-1}$)	Calibration Curves	r^2	Linear Range ng mL^{-1}	LOD	LOQ	Precision (% RSD, n = 6)		Repeatability (% RSD, n = 6)	Stability (% RSD, n = 6)	Recovery%					
							Intra-day	Inter-day			Low		Medium		High	
											Mean	RSD	Mean	RSD	Mean	RSD
Histidine	38.1	Y=546x-190000	0.9921	3.81 -38100	1.91	6.37	1.48	2.47	1.37	1.56	98.9	1.37	95.69	0.98	99.43	4.39
Glutamic acid	54.3	Y=65.3x+42500	0.9974	2.72-54300	2.72	9.07	1.02	2.89	1.6	1.23	96.4	1.76	96.87	4.02	100.53	4.26
Arginine	18.52	Y=1.31x-10300	0.9998	3.70 -4630	3.7	12.33	1.43	2.32	1.07	1.32	97.03	3.84	103.91	3.47	96.09	2.12
Cysteine	15.64	Y=63x-4200	0.9962	3.128-15640	3.13	10.43	2.51	2.97	2.0	2.91	99.05	4.87	97.91	2.30	102.34	2.11
Asparagine	14.6	Y=24.5x-2530	0.9997	2.92 -146000	2.92	9.73	1.72	1.91	1.59	2.31	97.96	4.02	98.98	1.73	103.90	4.39
Serine	19.84	Y=71.8x-5850	0.9998	3.968 -19840	3.96	13.2	1.21	2.43	1.32	2.69	98.8	3.00	98.92	3.23	103.75	1.82
Lysine	19.72	Y=402x-89100	0.9998	3.94-19720	3.94	13.13	1.32	1.6	1.43	1.2	95.39	4.75	99.27	4.80	98.32	2.57
Glutamine	18.2	Y=1540x-275000	0.9988	3.64 -18200	0.91	3.03	2.98	1.52	2.82	2.45	98.87	1.66	96.48	4.47	99.86	3.12
Proline	19.2	Y=699x-10900	0.9999	3.84-19200	0.96	3.2	1.67	1.06	1.39	2.15	97.73	4.39	98.55	1.48	103.11	2.08
Cytidine	30.7	Y=1500x-310000	0.999	6.14 -15350	1.54	5.13	2.41	2.93	2.13	1.36	100.1	3.33	97.82	1.85	98.46	2.02
Hypoxanthine	9.4	Y=10500x-42400	0.996	9.40 -9400	1.88	6.27	2.12	1.49	1.1	2.53	97.05	3.45	99.75	4.82	102.31	3.25
Dideoxycytidine	21.0	Y=1020x-13300	0.9986	4.20 -10500	1.05	3.5	2.68	2.66	2.82	2.74	95.88	2.76	99.42	1.66	98.01	4.12

Uridine	18.0	Y=81.6x-5880	0.9997	3.6-18000	1.8	6	1.98	2.71	2.21	1.45	96.88	3.65	100.64	1.20	102.06	3.32
Tyrosine	20.08	Y=761x-21100	0.9999	4.02-20080	4.02	13.4	1.74	2.58	1.11	3.2	99.9	2.12	98.62	2.16	97.05	2.41
Guanine	20.8	Y=151x-56200	0.9973	4.16 -20800	4.16	13.87	1.29	2.02	1.34	2.89	100.08	1.11	100.29	2.88	103.86	1.16
Guanosine	18.6	Y=647x-57200	0.9991	3.72-18600	1.86	6.2	2.13	2.59	2.63	2.61	98.59	1.50	95.56	4.90	99.62	1.57
Inosine	16.6	Y=7510x-39500	0.999	3.32-16600	1.66	5.53	2.68	1.96	2.25	2.23	99.84	1.61	96.95	3.11	97.41	1.87
Fumaric acid	3.8	Y=19.4x+16100	0.9991	7.6-3800	3.8	12.67	2.36	1.89	1.59	2.14	96.05	2.83	102.37	2.28	97.32	1.71
Dideoxyguanosine	13.44	Y=1120x-94300	0.9994	2.69-13440	2.69	8.97	1.77	2.53	2.62	3.01	99.32	4.27	98.32	4.09	98.13	4.90
Isoleucine	15.0	Y=150x+366	0.9994	15.00 -15000	3	10	3.05	1.59	1.85	3.54	101.0	1.87	98.51	2.68	97.49	1.66
Leucine	41.6	Y=53.9x+366	0.9998	8.32 -20800	2.08	6.93	1.87	1.49	2.76	2.88	100.86	3.44	97.8	2.77	103.71	4.41
Gallic acid	21.08	Y=57.3x-31500	0.9996	4.22 -10540	4.22	14.07	2.99	2.91	1.58	2.76	99.52	4.03	103.58	4.30	97.28	3.84
Thymidine	8.8	Y=160x-9940	0.9996	8.80 -8800	1.76	5.87	1.58	2.51	2.07	1.72	99.21	3.45	103.78	2.96	103.08	4.68
Phenylalanine	40.48	Y=3650x+228000	0.9999	8.10 -404800	2.02	6.73	1.47	2.85	2.95	1.35	98.31	2.33	100.68	2.61	95.80	1.04
Gallocatechin	20.48	Y=121x-110000	0.9992	4.10 -20480	4.1	13.67	1.15	2.11	1.37	1.91	100.76	4.18	95.56	3.51	99.95	4.93
Neochlorogenic acid	22.48	Y=69.1x-18200	0.9997	4.50 -22480	4.5	15	2.87	2.99	1.69	2.73	97.43	3.69	103.61	2.67	97.26	4.32
Tryptophan	13.0	Y=3310x+13500	0.9999	2.60 -6500	2.6	8.67	3.1	2.67	1.64	1.85	95.7	1.57	99.45	4.25	103.76	1.77
Epigallocatechin	22.04	Y=57.2x-24200	0.9999	22.04 -22040	1.1	3.67	1.35	1.41	2.38	1.91	103.4	3.86	103.10	4.25	96.97	1.87
Chlorogenic acid	61.52	Y=871x-121000	0.9999	6.15 -30760	6.15	20.5	2.28	3.25	1.66	1.33	98.66	2.97	103.62	1.64	96.84	3.42
Catechin	19.64	Y=13.7x-4980	0.9998	3.93 -19640	3.93	13.1	2.65	1.56	2.96	2.09	99.45	4.70	95.44	2.98	103.55	4.50
Cryptochlorogenic acid	21.6	Y=190x-44100	0.9994	4.32 -21600	4.32	14.4	3.3	2.34	2.54	1.52	95.29	4.10	100.53	1.13	100.10	1.80

Caffeic acid	18.44	Y=214x-102000	0.9922	3.69 -9220	3.69	12.3	2.54	1.79	2.21	1.32	97.65	1.73	97.48	2.45	95.30	2.58
Epicatechin	22.84	Y=368x-23900	0.9997	4.57<br>-228400	4.57	15.23	2.82	1.72	1.75	1.14	102.01	2.14	95.36	4.29	101.01	2.51
Rutin	20.2	Y=217x+4440	0.9999	4.04 -20200	1.01	3.37	1.56	2.18	1.67	2.41	101.86	1.79	97.59	4.30	95.19	1.83
Hyperoside	20.0	Y=197x+64100	0.9997	4.0-20000	4	13.33	1.25	1.64	2.9	2.79	99.94	2.62	100.77	3.83	100.70	2.91
Isoquercitrin	21.22	Y=226x+48000	0.9999	4.24 -21220	4.24	14.13	2.06	1.08	2.31	1.13	103.86	1.94	95.81	1.67	99.66	2.19
Quercitrin	18.66	Y=210x-99000	0.9999	18.66<br>-18660	3.73	12.43	1.59	1.51	1.52	1.28	99.45	4.60	102.56	4.10	98.21	1.85
Avicularin	19.26	Y=1920x-31900<br>0	0.9992	3.85-9630	3.85	12.83	2.14	1.84	1.29	2.32	96.99	3.55	97.51	2.80	96.51	3.87
Kaempferol 3-O- rutinoside	19.84	Y=361x-1150	0.9997	19.84-19840	3.97	13.23	1.92	2.37	1.89	1.68	96.22	2.51	100.14	1.91	98.61	2.57
Trifolin	12.04	Y=509x+68000	0.9999	12.04<br>-12040	2.41	8.03	0.87	2.58	2.67	2.99	95.89	4.89	103.40	4.24	96.44	3.60
Astragalin	23.58	Y=225x+56400<br>0	0.9991	4.72 -23580	4.72	15.73	2.23	2.85	1.85	2.25	99.00	4.98	98.26	2.80	102.81	1.27
Amentoflavone	20.22	Y=0.147x-4.85	0.9979	4.04 -10110	4.04	13.47	1.17	2.39	1.94	1.47	102.39	2.73	103.36	4.87	95.92	4.24
Apigenin	19.62	Y=0.473x+210	0.9921	3.92-19620	3.92	13.07	1.84	1.63	2.36	2.29	101.18	4.79	99.12	3.75	103.75	4.85

Table S4. Contents of 43 constituents in the AVF under salt stress

Treatments	Contents (μ g g^{-1}, *n*=3)									
	Histidine	Glutamic acid	Arginine	Cysteine	Asparagine	Serine	Lysine	Glutamine	Proline	Cytidine
0 mM	19.16±0.50a	76.02±2.00d	98.14±6.86ab	4.12±0.44c	706.38±0.71b	96.31±3.81a	104.98±17.02a	196.96±5.79b	44.69±1.94d	20.65±1.12b
100 mM	19.18±0.71a	100.10±9.17c	106.49±12.59a	4.62±0.00bc	716.05±1.09ab	101.29±0.75a	107.95±3.95a	207.57±2.40a	67.65±3.22b	28.77±0.95a
200 mM	18.90±0.08a	160.01±10.00a	90.23±5.55ab	5.12±0.51b	718.93±4.11a	89.56±0.87b	123.25±8.09a	176.95±2.70c	84.40±0.65a	26.28±1.03a
300 mM	18.89±0.08a	130.02±20.00b	81.04±3.1b	6.18±0.50a	713.23±8.07ab	73.03±3.79c	84.04±4.09b	144.73±7.36d	55.12±4.48c	29.15±2.81a

**Treatments**	Hypoxanthine	Dideoxycytidine	Uridine	Tyrosine	Guanine	Guanosine	Inosine	Fumaric acid	Dideoxyguanosine	Isoleucine	Leucine
0 mM	0.991±0.03b	0.805±0.10a	10.531±0.57c	114.971±8.28a	12.000±2.00c	3.589±0.28c	22.828±1.17a	30.667±10.41a	4.679±0.08b	36.338±1.66a	121.416±6.35a
100 mM	3.450±0.30a	0.886±0.07a	13.411±0.57a	126.943±15.15a	15.865±0.81b	5.348±0.35a	12.728±1.20c	37.000±4.36a	4.790±0.04b	45.964±5.09a	127.495±12.27a
200 mM	2.283±1.25a	0.909±0.07a	11.991±0.40b	115.075±8.17a	21.078±0.71a	5.287±0.10a	16.327±1.49b	50.304±26.70a	4.957±0.02a	48.382±3.93a	133.923±5.38a
300 mM	2.818±0.08a	0.894±0.10a	9.137±0.70d	78.122±55.31a	23.308±1.48a	4.502±0.24b	10.972±0.88c	46.000±3.46a	5.035±0.11a	51.246±8.50a	136.082±6.45a

Treatments	Gallic acid	Thymidine	Phenylalanine	Gallocatechin	Neochlorogenic acid	Tryptophan	Epigallocatechin	Chlorogenic acid	Catechin	Cryptochlorogenic acid	Caffeic acid
0 mM	30.10±2.01a	25.54±1.45a	78.82±3.44b	225.80±2.07d	8626.80±231.07a	206.36±1.21c	326.63±3.77d	892.30±4.39a	57.00±1.00b	741.46±88.38a	159.07±7.07b
100 mM	35.01±3.21a	16.78±0.69b	84.129±2.09b	305.31±5.16c	7275.88±107.87b	209.60±1.38c	493.15±10.70c	785.90±44.32b	59.70±4.52b	636.38±31.75a	145.17±6.12c
200 mM	50.02±7.78a	14.05±0.67c	82.22±6.05b	472.61±8.83a	6862.01±426.96bc	222.53±1.96b	617.46±3.30a	911.86±11.41a	77.94±1.89a	814.87±19.62a	176.30±5.77a
300 mM	40.03±9.17a	5.96±0.13d	95.54±6.87a	320.55±5.90b	6439.41±80.23c	242.36±2.24a	520.89±22.62b	868.72±22.03a	59.69±0.56b	827.02±111.65a	186.67±5.74a

Treatments	Epicatechin	Rutin	Hyperoside	Isoquercitrin	Quercitrin	Avicularin	Kaempferol 3-O-rutinoside	Trifolin	Astragalin	Amentoflavone	Apigenin
0 mM	139.27±9.83c	52.81±13.07a	2150.67±350.44b	3109.43±164.10b	25.53±0.59a	15.33±0.51b	38.72±0.91d	312.67±35.48ab	360.51±20.93ab	299.90±0.02a	466.97±31.14a
100 mM	271.53±27.13b	54.21±8.50a	2580.05±127.24b	3581.24±133.45a	25.85±0.13a	20.82±1.61a	83.48±6.46c	366.58±22.11a	398.42±11.53a	318.18±18.29a	515.28±45.49a
200 mM	319.93±6.97a	74.05±5.49a	2996.03±66.02a	3225.90±34.83b	25.50±0.23a	19.37±2.28a	141.93±2.02b	333.97±29.45a	346.07±17.83b	322.63±12.78a	511.06±81.93a
300 mM	287.63±15.60b	61.85±0.59a	1790.79±125.47c	2835.59±86.73c	25.75±0.14a	13.12±1.16b	169.91±21.99a	261.16±26.19b	289.63±29.26c	306.01±10.58a	496.05±25.14a

The four groups were the same as in Figure 2. Data are the mean ± SD (n = 3). Different letters following values in the same column indicate significant difference among salt treatments using Duncan's multiple-range test at $p < 0.05$

Figure S4 Chemical structures of 43 compounds analyzed in this study

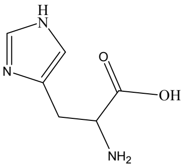

1. Histidine

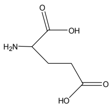

2. Glutamic acid

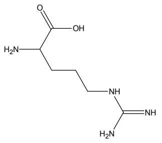

3. Arginine

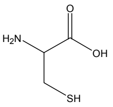

4. Cysteine

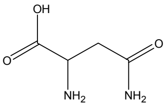

5. Asparagine

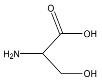

6. Serine

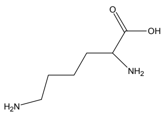

7. Lysine

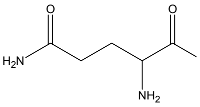

8. Glutamine

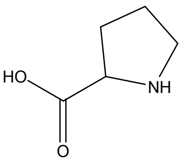

9. Proline

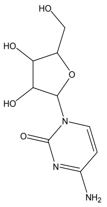

10. Cytidine

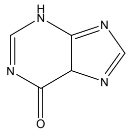

11. Hypoxanthine

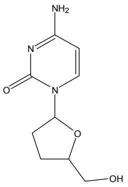

12. dideoxycytidine

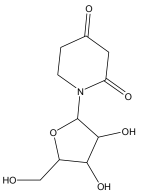

13. Uridine

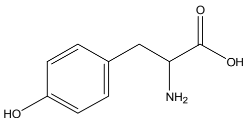

14. Tyrosine

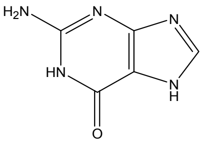

15. Guanine

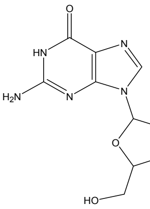

16. Guanosine

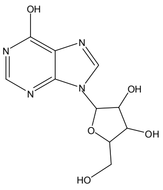

17. Inosine

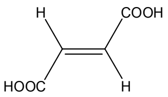

18. Fumaric acid

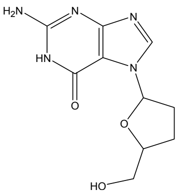

19. dideoxyguanosine

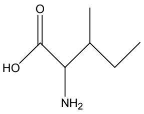

20. Isoleucine

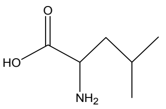

21. Leucine

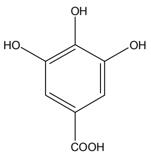

22 Gallic acid

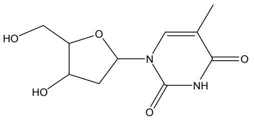

23. Thymidine

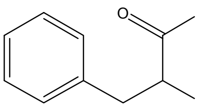

24. Phenylalanine

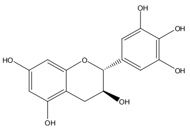

25.Gallocatechin

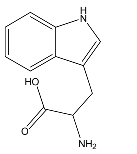

27. Tryptophan

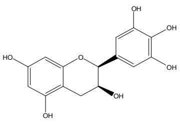

28. Epigallocatechin

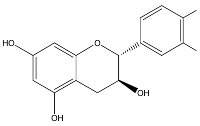

30. Catechin

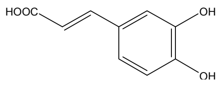

32. Caffeic acid

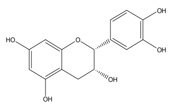

33. Epicatechin

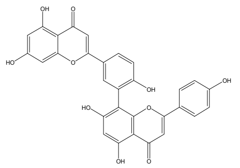

42. Amentoflavone

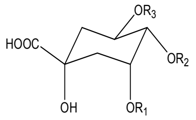

26. Neochlorogenic acid:R_1=R_2=H,R_3=caffeoyl

29. Chlorogenic acid: R_1=caffeoyl, R_2=R_3=H

31. Cryptochlorogenic acid:R_1=R_3=H,R_2=caffeoyl

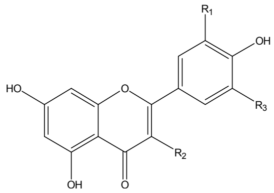

34. Rutin: R_1=OH, R_2=O-rutinose,R_3=H

35. Hyperoside: R_1=OH, R_2=O-galactose,R_3=H

36. Isoquercetin: R_1=OH, R_2=O-glucose,R_3=H

37. Quercitrin: R_1=H, R_2=O-arabinose,R_3=OH

38. Avicularin: R_1=OH,R_2=O-arabinose,R_3=H

39. Kaempferol 3-O-rutinoside: R_1=H, R_2=O-galactose,R_3=H

40. Trifolin: R_1=H, R2=O-galactose,R_3=H

41. Astragalin: R_1=H, R_2=O-glucose,R_3=H

43. Apigenin: R_1=H, R_2=H,R_3=H
